# Interferon-α resistance in renal carcinoma cells is associated with defective induction of signal transducer and activator of transcription 1 which can be restored by a supernatant of phorbol 12-myristate 13-acetate stimulated peripheral blood mononuclear cells

**DOI:** 10.1038/sj.bjc.6600066

**Published:** 2002-02-01

**Authors:** A Brinckmann, S Axer, D Jakschies, I Dallmann, J Grosse, T Patzelt, T Bernier, A Emmendoerffer, J Atzpodien

**Affiliations:** Department of Hematology and Oncology, Medizinische Hochschule, Hannover, Germany; Fraunhofer Institut, Hannover, Germany; European Institute for Tumor-Immunology and Prevention (EUTIP), 152 Gotenstr., 53175, Bonn, Germany; Fachklinik Hornheide of the University of Münster, Münster-Handorf, Germany

**Keywords:** cytokine, signal transduction pathway, Jak, cancer therapy

## Abstract

Therapy of selected human malignancies with interferon-α is widely accepted but often complicated by the emergence of interferon-α resistance. Interferon is a pleiotropic cytokine with antiproliferative, antitumour, antiviral and immunmodulatory effect; it signals through the Jak-STAT signal transduction pathway where signal transducer and activator of transcription 1 plays an important role. Here we report both, a lack of signal transducer and activator of transcription induction in interferon-α resistant renal cell carcinoma cells and signal transducer and activator of transcription 1 reinduction of phorbol 12-myristate 13-acetate-stimulated peripheral blood mononuclear cells supernatant. Preliminary experiments on the identification of the molecules that reinducing signal transducers and activators of transcription 1 indicate that interferon-γ may be the responsible candidate cytokine, but several others may be involved as well. This work provides the basis for therapeutic strategies directed at the molecular modulation of interferon-α resistance in human neoplasms.

*British Journal of Cancer* (2002) **86**, 449–455. DOI: 10.1038/sj/bjc/6600066 www.bjcancer.com

© 2002 The Cancer Research Campaign

## 

Interferon-α (IFN-α) plays an important role in the treatment of various human malignancies, among them renal cell carcinoma (Dorr, 1993); however, response to IFN-α is often impaired by the development of IFN-resistance (Devita *et al*, 1989), mechanisms of which are poorly understood.

Interferon-α belongs to a group of cytokines with antiviral, antiproliferative, antitumour and immunmodulatory activities (Pestka *et al*, 1987). Binding of IFN-α to the IFN Type I receptor results in oligomerization of the receptor subunits and subsequent transphosphorylation of receptor-associated Janus-kinases Jak1 and Tyk2; activated Jak1 and Tyk2 subsequently phosphorylate tyrosine residues on the associated receptor chain. Signal transducers and activators of transcription (STAT) 1 and 2 can then bind to the receptor by their SH2 domains which are thereupon tyrosine phosphorylated by the receptor-associated Janus-kinases; thereafter, the STATs are released from the receptor and form STAT1-STAT2-heterodimers which translocate to the nucleus where they bind with p48 to form the interferon stimulated gene factor 3 (ISGF3). ISGF3 binds to the interferon stimulated response element (ISRE) in the promoter of IFN-induced genes resulting in transcription of interferon-stimulated genes (ISG) (Schindler and Darnell, 1995; Haque and Williams, 1998).

There is evidence that IFN-α resistance is associated with defective components of the Jak-STAT-Pathway (Pansky *et al*, 2000) e.g., defective activation of ISGF3 (Xu *et al*, 1994; Wong *et al*, 1997), lack of STAT1 expression (Sun *et al*, 1998) or STAT3 induction (Yang *et al*, 1998). It has been reported that sequential treatment of interferon resistant cells with retinoic acid or tamoxifen followed by interferon-α up-regulates STAT1 expression and ISGF3 activation, respectively, in cells which do not respond to either single agent (Kolla *et al*, 1996; Lindner *et al*, 1997).

Here we sought (a) to characterize STAT1 deficiency associated with IFN-α resistance and (b) to identify modulators of STAT1 induction. These studies provide the basis for a potential modulation of resistance to IFN-α in renal cell carcinoma *ex-vivo* as well as in renal cell carcinoma patients receiving systemic IFN-α.

## MATERIALS AND METHODS

### Cells and culture conditions

A-498 and Caki-2 cells were obtained from ATCC (Rockville, USA) (Cat. No. HTB 44). The fresh RCC cells were established according to Ebert *et al* (1990). Cells were grown in RPMI 1640 supplemented with 10% heat-inactivated FCS, 2 mM L-glutamine, 50 mg ml^−1^ streptomycin and 50 IU ml^−1^ penicillin at 37°C in an atmosphere containing 5% CO_2_ and passaged once a week.

### Reagents

Interferon-α-2a (Roferon A® (18×10^6^ IU), Hoffmann-LaRoche, Basel, Switzerland) was dissolved in Aqua bidest, diluted with RPMI 1640 and stored in aliquots of 1×10^6^ IU at 4°C. IFN-α was used in a final concentration of 1000 IU ml^−1^. IFN-γ-1b (Imukin® (3×10^6^ IU)) was obtained from Boehringer Ingelheim, dissolved in Aqua bidest and stored in aliquots of 10 ng ml^−1^ (300 IU ml^−1^) at 4°C. Phorbol 12-myristate 13-acetate (PMA) was obtained from Sigma. It was dissolved in DMS and stored as aliquots (10 μg ml^−1^) at 4°C.

### Interferon*-α-*2a dose titration

Cells were tested for IFN-α resistance using different IFN-α-2a concentrations (10, 100, 1000, 10 000 IU ml^−1^) in the Cell Proliferation ELISA, BrdU (colorimetric), (Roche Molecular Biochemicals, Mannheim, Germany).

A relative resistance to the antiproliferative effects of interferon-α was best produced by continuous incubation of the A-498 and fresh renal cell carcinoma (RCC) cells with 1000 IU ml^−1^ interferon-α over 3–4 months.

### Preparation of PMA-stimulated PBMC supernatant

Peripheral blood mononuclear cells (PBMC) were isolated from buffy-coat leucocyte concentrates of healthy donors by Ficoll gradient centrifugation. The cells were cultured in RPMI 1640 supplemented as indicated above and stimulated with 10 ng ml^−1^ PMA for 4 days. After centrifugation for 10 min at 225 **g** the media was decanted and again centrifuged for 10 min at 2000 **g**. The supernatant was diluted 1 : 2 with RPMI 1640 and added to the cells.

### Whole cell extracts

**Figure 1:**
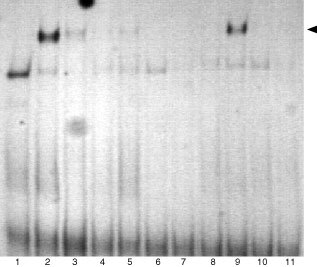
Electrophoretic mobility shift assays for IFN-α induced STAT1 induction. IFN-α sensitive (S) and IFN-α resistant (R) A-498 and fresh RCC cells and Caki-2 cells were either left untreated or incubated with 1000 IU ml^−1^ IFN-α for 30 min. Lanes 1–5 are A-498 cells: lanes 1 and 4, untreated IFN-α sensitive and resistant cells, respectively; lanes 2, 3 and 5, IFN-α stimulated IFN-α sensitive and resistant cells, respectively. Lanes 6 and 7 are Caki-2 cells: lane 6, untreated Caki-2 cells; lane 7, IFN-α stimulated cells. Lanes 8–11 are fresh RCC cells: lanes 8 and 10, untreated IFN-α sensitive and resistant cells, respectively; lanes 9 and 11, IFN-α stimulated IFN-α sensitive and resistant cells, respectively. STAT1 induction can be detected in sensitive A-498 and fresh RCC cells after stimulation with IFN-α but not in resistant cells (lanes 2 and 9). STAT1 can be competed with monoclonal anti-STAT1 antibody (lane 3) (see arrow for STAT1 band).

Cells were treated with IFN-α, IFN-γ and supernatant as single agents, as well as with combinations of IFN-α and supernatant for various times as indicated in Figures 1

, 2

**Figure 2:**
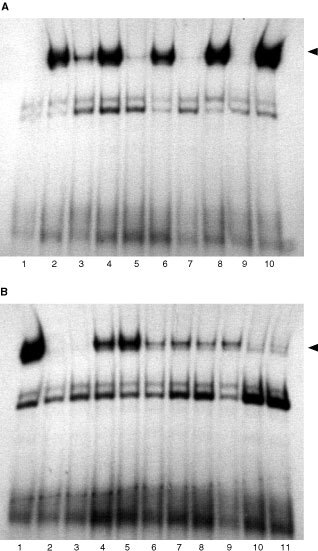
Electrophoretic mobility shift assays for STAT1 induction in A-498 cells by PMA-stimulated PBMC supernatant. (**A**) Electrophoretic mobility shift assay for STAT1 induction in IFN-α sensitive (S) A-498 cells by PMA-stimulated PBMC supernatant. Cells were either left untreated (lane 1) or treated with IFN-α alone (lane 2) or supernatant alone (lanes 3, 5, 7, 9) or with their combination (lanes 4, 6, 8 and 10). STAT1 is induced in the presence of IFN-α (lanes 2, 4, 6, 8 and 10) and after incubation for 1 h with the supernatant (lane 3) (see arrow for STAT1 band). (**B**) STAT1 induction in IFN-α resistant (R) A-498 cells by PMA-stimulated PBMC supernatant. Cells were treated with IFN-α alone (lane 3) or supernatant alone (lanes 4, 6, 8 and 10) or with their combination (lanes 5, 7, 9 and 11). IFN-α does not induce STAT1 (lane 3) but treatment with supernatant alone and in combination with IFN-α for 1, 4 and 6 h can induce STAT1 induction (lanes 4–9). IFN-α treated A-498 S cells served as positive control (lane 1) (see arrow for STAT1 band).

, 3

**Figure 3:**
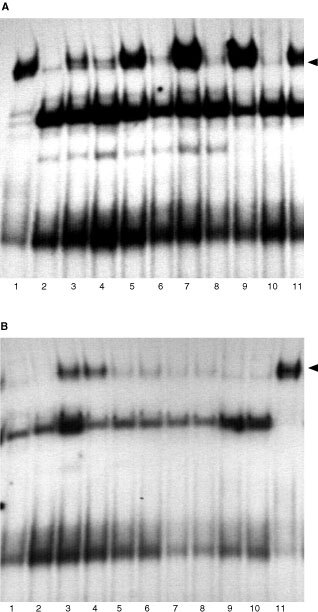
Electrophoretic mobility shift assays for STAT1 induction in fresh RCC tumour cell culture by PMA-induced PBMC supernatant. (**A**) STAT1 induction in IFN-α sensitive (S) fresh RCC tumour cell culture by PMA-induced PBMC supernatant. Cells were either left untreated (lane 2) or treated with IFN-α alone (lane 3) or supernatant alone (lanes 4, 6, 8 and 10) or with their combination (lanes 5, 7, 9 and 11). STAT1 is induced in the presence of IFN-α (lane 3, 5, 7, 9 and 11) and after incubation for 1 h with the supernatant (lane 4). IFN-α treated A-498 S cells served as positive control (lane 1) (see arrow for STAT1 band). (**B**) STAT1 induction in IFN-α resistant (R) fresh RCC cells by PMA-stimulated PBMC supernatant. Cells were either left untreated (lane 1) or were treated with IFN-α alone (lane 2) or supernatant alone (lanes 3, 5, 7 and 9) or with their combination (lanes 4, 6, 8 and 10). IFN-α did not induce STAT1 (lane 2) but treatment with supernatant alone and in combination with IFN-α for 1 h induced STAT1 induction (lanes 3 and 4). IFN-α treated A-498 S cells served as positive control (lane 11) (see arrow for STAT1 band).

, 4

**Figure 4:**
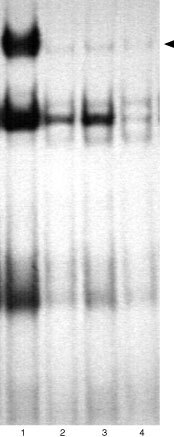
Electrophoretic mobility shift assay for STAT1 induction in Caki-2 cells by PMA-induced PBMC supernatant. Lanes 2–4 are Caki-2 cells: Lane 2 are untreated Caki-2 cells. Cells were treated with supernatant alone (lane 3) or with IFN-α and supernatant (lane 4). There is no STAT1 induction detectable in Caki-2 cells. IFN-α treated A-498 S cells served as positive control (lane 1) (see arrow for STAT1 band).

, 5

**Figure 5:**
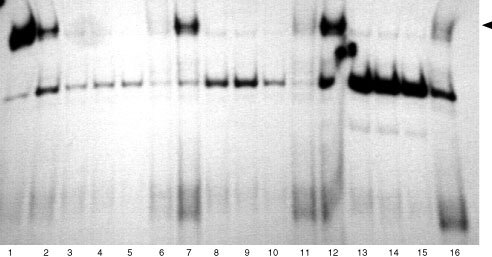
Confirmation of supernatant-induced STAT1 via competition with STAT1-mAb and unlabelled oligo. Supernatant-induced STAT1 induction in IFN-α sensitive (S) A-498 cells (lane 2) and IFN-α resistant (R) A-498 cells (lanes 7 and 12) can be competed with STAT1-mAb (lanes 6, 11 and 16) or 50-, 100- and 300-fold excess of unlabelled oligo (lanes 3–5, 8–11, 13–15) (see arrow for STAT1 band).

and 7

**Figure 7:**
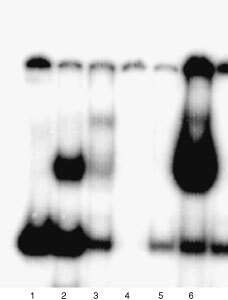
Sensitive (S) cells were either left untreated (lane 1) or were treated with IFN-α alone (lane 2) or with the combination of IFN-α and a monoclonal STAT1 antibody (lane 3). Resistant (R) cells were either left untreated (lane 4) or were treated with IFN-α alone (lane 5) or with IFN-γ alone (lane 6). IFN-α fail to induce STAT1 in resistant cells (but it was possible to induce STAT1 in the sensitive cells). The signal extinguished in sensitive cells when a commercially available monoclonal STAT1 antibody was added (lane 3). In contrast IFN-γ was able to reinduce STAT1 in IFN-α resistant cells (lane 6). Untreated A-498 S and R cells were used as negative controls (lanes 1 and 4).

. IFN-α or IFN-γ was added during the last 30 min of incubation with supernatant. Cells were washed with ice-cold phosphate-buffered saline (PBS) and resuspended in RSB buffer (10 mM Tris (pH 7.4); 10 mM NaCl; 1.5 mM MgCl_2_; 10 mM NaF; supplemented with 0.15 mM PMSF; 1 mM DTT; 0.2 mM Na_3_VO_4_) and incubated 10 min on ice for swelling. After centrifugation at 15 800 **g** for 10 s at 4°C, cells were resuspended in buffer C (20 mM HEPES; 420 mM NaCl; 1.5 mM MgCl_2_; 0.2 mM EDTA; 25% Glycerin; 10 mM NaF; supplemented with 0.15 mM PMSF; 1 mM DTT; 0.2 mM Na_3_VO_4_), sucked up and down 5–10 times in a 26 G syringe and incubated on ice for 45 min. Whole cell extract was obtained by centrifugation at 15 800 **g** for 10 min at 4°C. The supernatant (=whole cell extract) was shock frozen in liquid nitrogen at −175°C and stored at −80°C until use.

### Electrophoretic mobility shift assay (EMSA)

As STAT1 binding site we used the β-Casein (5′-TCGAAGATTTCTAGGAATTCAATC-3′) or FcγRI-GAS (5′-TCGATTTGAGATGTATTTCCCAGAA-3′). The oligos were synthesized at Roth, Karlsruhe, Germany and labelled at the 5′-end with ^32^P. Briefly whole cell extracts of equal protein concentration were incubated with the labelled oligo in 200 mM KCl; 2 mM MgCl_2_; 0.5 mM EGTA; 2.5 mM DTT; 100 mM HEPES (pH 7.9); 50% Glycerine and Poly-d(I-C) for 30 min at room temperature. For competition assays 1 μl monoclonal STAT1-antibody (Santa Cruz Biotechnology, Inc.) or 50-, 100- and 300-fold excess of unlabelled oligo were used. Electrophoresis was performed on a 6% non-denaturating polyacrylamide gel at 300 V for 15 min and 380 V for 2 h in the cold. Gels were dried and exposed to X-Omat AR 5 films (Kodack, Stuttgart, Germany) for 1 to 3 days.

### STAT1-reinduction experiments of* γ*-interferon

Based on experiments of Venkataraman *et al* (1999) IFN-γ-1b was used in a concentration of 10 ng ml^−1^. Experiments were performed in duplicate.

### Statistical analysis

Assays were regularly performed in triplicates and statistical means were established.

## RESULTS

### IFN*-α* associated antiproliferative effect and STAT1 induction in RCC cells

The RCC cell lines A-498 and Caki-2 and fresh RCC cells were assessed for the antiproliferative effect of IFN-α by a cell proliferation ELISA based on BrdU incorporation. In A-498 and fresh RCC cells, IFN-α inhibited cell proliferation in a dose-dependent manner while in Caki-2 cells, IFN-α showed no antiproliferative effect (data not shown).

As IFN-α is known to induce STAT1 in various cell types, cells were investigated for STAT1 induction. Cells were incubated for 30 min with 1000 IU ml^−1^ interferon-α and assessed for STAT1 induction by EMSA. We found that in A-498 and fresh RCC cells IFN-α induced STAT1 but not in Caki-2 cells, suggesting that primary resistance towards the antiproliferative effect of IFN-α is associated with defective STAT1 induction (Figure 1).

Identity of STAT1 was confirmed by adding STAT1-mAb which selectively blocked the STAT1-band.

### IFN-*α* resistant A-498 and RCC cells are defective in the induction of STAT1

IFN-α resistance of A-498 and fresh RCC cells, respectively, was induced by culturing the cells over 3–4 months in 1000 IU ml^−1^ IFN-α. A relative resistance to IFN-α in A-498 and fresh RCC cells was subsequently confirmed by cell proliferation ELISA.

An EMSA for determination of STAT1 activity was performed as indicated above. IFN-α failed to induce STAT1 induction in resistant A-498 and fresh RCC cells, but not in IFN-α sensitive A-498 and fresh RCC cells, respectively (Figure 1).

### PMA-stimulated PBMC supernatant can reinduce STAT1 in IFN-*α* resistant A-498 and fresh RCC cells

The supernatant was prepared as described below. A-498 cells were treated for 1, 4, 6 and 24 h either with the supernatant alone or with supernatant followed by 30 min 1000 IU ml^−1^ interferon-α as indicated in Figure 2A,B.

In the sensitive cells, the supernatant alone induced a visible band after 1 h of incubation, only. In the presence of IFN-α, a STAT1-band was detectable at all time points (Figure 2A). The supernatant alone and its combination with IFN-α were able to restore STAT1 induction in resistant cells. The most intense STAT1-band was detected after 1 h of stimulation, it was significantly less intense 4 and 6 h and disappeared at 24 h (Figure 2B). When repeated with the PBMC supernatant of another donor, this assay led to the identical results (data not shown).

As we sought to exclude a cell line specific effect of the supernatant, we treated fresh RCC cells in the same manner. These cells were first derived from one RCC patient, and an IFN-α resistant variant which also lacks STAT1 induction was subsequently generated.

In sensitive cells, the supernatant alone induced a weak STAT1-band after only 1 h. As expected in the presence of interferon-α, STAT1 was induced at all time points (Figure 3A). The present supernatant reinduced STAT1 in the IFN-α resistant cells with a clearly detectable band after 1 h (Figure 3B); Interferon-α treated A-498 S cells served as positive control for STAT1.

### In Caki-2 cells, PMA-stimulated PBMC supernatant cannot reinduce STAT1

As the supernatant induced optimal STAT1 in A-498 and fresh RCC cells after 1 h, Caki-2 cells were treated for 1 h with supernatant alone or in combination with IFN-α. Neither the supernatant alone nor its combination with interferon reinduced STAT1 in Caki-2 cells (Figure 4).

### Confirmation of supernatant-induced STAT1 via competition with STAT1-mAb and unlabelled oligo

To confirm that the band induced by the present supernatant correlated to STAT1, a competitional assay was performed with STAT1-mAb and with 50-, 100- and 300-fold excess of unlabelled oligo, which all blocked the STAT1-band (Figure 5).

### PMA alone or in combination with interferon-*α* does not induce STAT1

**Figure 6:**
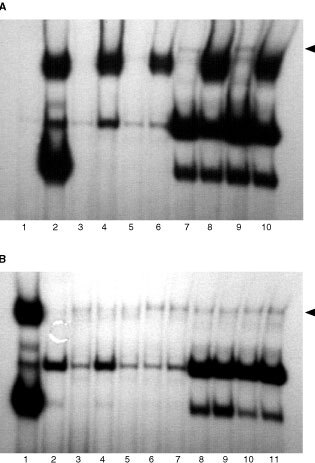
Electrophoretic mobility shift assays for STAT1 induction by 10 ng ml^−1^ PMA. (**A**) STAT1 induction in IFN-α sensitive (S) A-498 cells by 10 ng ml^−1^ PMA. Cells were either left untreated (lane 1) or were treated with IFN-α alone (lane 2) or PMA alone (lanes 3, 5, 7 and 9) or with their combination (lanes 4, 6, 8 and 10). STAT1 indiction can be detected after incubation with IFN-α alone (lane 2) and in combination with PMA (lanes 4, 6, 8 and 10). PMA alone does not induce STAT1 (lanes 3, 5, 7, 9) (see arrow for STAT1 band). (**B**) STAT1 induction in IFN-α resistant (R) A-498 cells by 10 ng ml^−1^ PMA. Cells were either left untreated (lane 2) or were treated with IFN-α alone (lane 3) or PMA alone (lanes 4, 6, 8 and 10) or with their combination (lanes 5, 7, 9 and 11). IFN-α and PMA alone as well as their combination fail to induce STAT1 in resistant cells. IFN-α treated A-498 S cells were used as positive control (lane 1) (see arrow for STAT1 band).

In order to demonstrate that the effect of STAT1 reinduction was due to the cytokines secreted by PMA-stimulated PBMC and not by PMA itself, A-498 cells were incubated with 10 ng ml^−1^ of PMA either alone and in combination with IFN-α. PMA alone did not induce STAT1 neither in the sensitive nor in the resistant cells, while the combination of PMA and IFN-α induced STAT1 in the sensitive cells, but not in the resistant cells (Figure 6A,B

).

### IFN-*γ* alone does induce STAT1

To investigate the cytokine potentially capable of STAT1 reinduction, IFN-α resistant A-498 cells were incubated with IFN-γ 1b (10 ng ml^−1^), which clearly reinduced STAT1. A-498 sensitive and resistant cells treated with IFN-α were used as positive and negative control for STAT1 (Figure 7).

## DISCUSSION

Response to IFN-α is often impaired by the development of IFN-resistance (Devita *et al*, 1989). Many mechanisms have been proposed to mediate IFN-α resistance in various different cell types: lack of ISGF3 (Xu *et al*, 1994; Wong *et al*, 1997) or one of its components (Dron and Tovey, 1993; Sun *et al*, 1998), decrease in ISG expression (Affabris *et al*, 1983; Salzberg *et al*, 1983), lack of a high affinity receptor site (Hannigan *et al*, 1984) or the development of anti-interferon antibodies in patients (Quesada *et al*, 1985; Steis *et al*, 1988). However, there seems to be no unique mechanism for all cells given reports on interferon-resistant cells which do not inherit one of the above defects (Grups and Bange, 1990; Talpaz *et al*, 1992). While an interferon-resistant cell line with entire STAT1 induction has been reported (Yang *et al*, 1998), STAT1 seems to be an essential component of interferon signalling as STAT1 deficient mice do not respond to interferons and are highly sensitive toward viral infections (Durbin *et al*, 1996; Meraz *et al*, 1996); in addition complementation of the IFN-unresponsive mutant cell line U3 interferon with STAT1, cDNA constructs reportedly restores ISGF3 formation and transcriptional response to interferon (Müller *et al*, 1993).

Here, we detected a deficient STAT1 induction in two different RCC sublines, with secondary interferon-α resistance and in a primary resistant RCC cell line. There were several possibilities to explain the defect, e.g.: (a) decreased synthesis, increased degradation, decreased tyrosine phosphorylation of STAT1; (b) malfunction in the preceding steps of the signal transduction pathway; or (c) lack of STAT1 transcript due to a mutation in the STAT1 gene.

In the present experiments, the PMA-stimulated PBMC supernatant reinduced STAT1 in A-498 and fresh RCC cells with secondary IFN-α resistance but not in primary resistant Caki-2 cells. This indicated that in resistant A-498 and fresh RCC cells, the STAT1 gene is still intact while in primary resistant Caki-2 cells, the defect in the signal transduction pathway appears to be more extensive. Previously, Sun *et al* (1998) reported a lack of STAT1 transcript in IFN resistant lymphoma cells. Here a mutation in or a loss of the STAT1 gene could explain why Caki-2 cells were refractory to PMA-PBMC-mediated STAT-reinduction.

It is known that PMA-stimulated PBMC produce a number of cytokines; as observed there are several cytokines including IL-2, IL-6, IL-9, IL-10, IL-11, IFN-γ, hormones as growth hormone and prolactin and growth factors as GCSF, EGF, PDGF, CSF-1 and angiotensin which may induce STAT1 in various cell types (Schindler and Darnell, 1995; Briscoe *et al*, 1996; Ihle, 1996; Leaman *et al*, 1996).

Here, we demonstrated that IFN-γ reinduces STAT1 in IFN-α resistant A-498 cells; this suggested that IFN-γ may be the PMA-stimulated PBMC derived cytokine capable of reinducing STAT1 but several others may be involved as well. Since biological effects of IFN-α and IFN-γ are both mediated by Jak1, it appeared that Tyk2 tyrosine kinase or the receptor may be impaired in IFN-α resistant carcinoma cells upstream of STAT1.

The significance of the additional STAT1 inducers remained unclear as their biological effects are not necessarily mediated by a Jak-STAT signal transduction pathway or require STAT1 induction. Previously, STAT1 deficient mice responded normally to growth hormone, EGF and IL-10 which induce STAT1 *in vitro* (Meraz *et al*, 1996). STAT1 induction by cytokines other than interferons were cell-specific (Schindler and Darnell, 1995; Han *et al*, 1996). Therefore, in RCC some of these known cytokines may actually not be able to induce STAT1, while there might be additional inducers.

Future experiments will focus on further steps toward the identification of those molecular regulators associated with IFN-α resistance in order to provide new modalities of treatment for renal cell carcinoma patients.
